# Predictors of enrollment in a health protection scheme among informal sector workers in Kumasi Metropolis of Ghana

**DOI:** 10.1186/s13104-019-4782-2

**Published:** 2019-11-21

**Authors:** Dina Adei, Williams Agyemang-Duah, Anthony Acquah Mensah

**Affiliations:** Department of Planning, Kwame Nkrumah University of Science and Technology, Kumasi, Ghana

**Keywords:** National Health Insurance Scheme, Enrollment, Informal sector workers, Kumasi Metropolis

## Abstract

**Objective:**

Informal sector workers are exposed to occupational hazards which could escalate their healthcare expenditures. Thus, enrollment in a health protection scheme among informal sector workers is useful for reducing their catastrophic healthcare expenditures. However, there is scant information on factors predicting their enrollment in the National Health Insurance Scheme (NHIS) in Ghana, a gap this quantitative study aims to fill. A sample of 350 informal sector workers was involved in a cross-sectional survey.

**Results:**

Approximately 17% of the participants were enrolled in NHIS. Respondents who had worked between 5 and 7 years were significantly more likely to enroll in NHIS compared with those who had worked below 2 years **(**AOR = 13.159, CI 1.135–152.596, p = 0.039). The study further found that apprentices (AOR = 0.72, CI 0.353–1.056, p = 0.005) were less likely to enroll in NHIS compared with their masters. Participants who were exposed to electrical hazards (AOR = 2.93, CI 1.56–5.10, p = 0.013) and suffered from occupational diseases (AOR = 2.75, CI 1.743–5.17, p = 0.001) were significantly more likely to enroll in NHIS. Also, respondents who were non-Christians were significantly less likely to enroll in NHIS compared with their respective counterparts (AOR = 0.726, CI 0.067–2.503, p = 0.011). The findings are useful for increasing the NHIS enrollment rate among informal sector workers in Ghana.

## Introduction

After independence, the Government of Ghana introduced free healthcare for its citizens [1], with the rationales of removing all forms of barriers to healthcare accessibility and utilization and, also ensuring universal health coverage [2]. Free healthcare was funded through taxes and donor support [3]. As part of the free healthcare policy, the government provided additional healthcare facilities across the country and strengthened preventive interventions such as immunization and antenatal care. In the 1980s, however, due to the poor economic growth resulting in limited resources, the government abolished the free healthcare policy [2].

Thus, the out-of-pocket payment systems were gradually implemented in Ghana in the mid-1980s [4]. With this policy, there was a total withdrawal of government subsidy with patients taking care of their healthcare expenditures. The justifications of this policy were to increase funds for providers, make fee recovery legal and restrict unnecessary utilization of healthcare [2]. However, the policy was serving as an obstacle to healthcare utilization [5]. For instance, outpatient visits reduced by 66% as a result of the implementation of the cash and carry system [2].

The National Health Insurance Scheme (NHIS) was introduced in 2004 as an alternative healthcare financing mechanism in Ghana [6]. The NHIS provides free enrollment for Social Security and National Insurance Trust (SSNIT) retirees, persons aged 70 years or older, pregnant women, children under 18 years and paupers [7]. It covers about 95% of disease burden conditions of people [8] including malaria, diarrhea, respiratory tract infections, skin diseases, hypertension, diabetes and asthma [9].

The national enrollment rates of NHIS in 2010, 2015 and 2017 were 33% (8.2 million), 41% (11.3 million) and 35% (10.3 million) respectively. With this, the enrollment rate of NHIS among informal sector workers in 2017 was 29.8% [6], which is below the 2017 national enrollment rate. This shows that informal sector workers have a low enrollment rate in NHIS despite their frequent exposure to occupational hazards [10] such as physical, chemical, biological [11] and other occupational injuries [12]. As such, they are likely to incur catastrophic healthcare expenditures due to their frequent exposure to occupational hazards coupled with their low NHIS enrollment. Enrollment in NHIS could help to minimize their healthcare expenditures [13]. Evidence shows that socio-demographic factors [14], such as education, gender, and income are associated with NHIS enrollment [15]. However, there is scant information on factors influencing NHIS enrollment among informal sector workers in Ghana, a gap this quantitative study aims to fill. Understanding this knowledge area would be useful for increasing the NHIS enrollment rate among informal sector workers in Ghana.

## Main text

### Methods

#### Study settings

The study was conducted in Kumasi Metropolis of Ghana. Kumasi Metropolis represents the most populous settlement in Ghana with a population of 1,730,249 inhabitants which comprised 47.8% males and 52.2% females. About 60% of the labor force in Kumasi Metropolis predominantly engaged in informal economic activities [16].

##### Sample and data

This study was a cross-sectional survey. A headcount of all potential participants was undertaken from September to November 2016 to determine the sampling frame because of the absence of data on the population. The headcount covered the masters and apprentices working as welders and fish processors within the study area. The headcount identified a total of 200 fish processors and ten master welders at Asafo. Meanwhile, 180 master welders were identified at Suame magazine adding up to a total of 190 welders. Slovin’s sample size determination method [17], was used to determine the sample size.

$${\text{n}} = {{\text{N}} / {\left[ {1 + {\text{N}}\left( \upalpha \right)^{2} } \right]}}$$

where, n = sample size; α = error margin of 0.05 and a confidence interval of 95%, and N = the sampling frame. The formula was applied separately for fish processors and master welders and a sample size of 262 (129 master welders and 133 fish processors) was obtained. For every master welder selected, his senior apprentice was also selected adding up to a total of 258 welders (129 masters and 129 apprentices) and 133 fish processors yielding a total sample size of 391 participants. The fish processors had no apprentices. The response rate was 97.7% for fishmongers and 85.3% for welders, a total response rate of 91.5%. Therefore, the sample size used for analyses was 350 participants comprising 220 welders and 130 fish processors. The simple random sampling was used to select the respondents because it is mostly used in health research [18]. This was operationalized by writing the numbers on pieces of paper, keeping them in a box and shuffling thoroughly. The papers were drawn one after the other until the required sample size was obtained.

Face-to-face interviewer-administered questionnaires covering information on socio-demographic, medical characteristics and enrollment in NHIS were used to elicit data from the respondents. The questions were in English but were translated to Twi (a local language of the participants) for easy understanding. This was therefore converted back to English for analyses. Written and verbal informed consents were sought from the respondents before data collection. Respondents were further assured of strict confidentiality of the information they provided.

##### Measures

*Outcome variable* The outcome variable was enrollment in NHIS and was measured as a dichotomous variable indicating ‘yes = 1’ or ‘no = 0’.

*Predictor variables* The independent variables were type of enterprise (1 = welding, 2 = fish processing), type of respondents (1 = master, 2 = apprentices, 3 = employee), gender (1 = male, 2 = female), age (years) (1 = 49 or below 2 = 50 or above), education (1 = no formal education, 2 = basic, 3 = secondary, 4 = tertiary), marital status (1 = married, 2 = single), religion (1 = Christians, 2 = non-Christians), work experience (years) (1 = below 2, 2 = 2–4, 3 = 5–7, 4 = 8–10, 5 = 11–13, 6 = above 13), income (1 = GH¢ < 1000, 2 = 1000 or above). Membership of workers association, exposure to welding light, biological hazards, electrical hazards, fire hazards, occupational injury and occupational diseases were measured as ‘yes = 1’ or ‘no = 0’.

##### Analytical framework

Data were entered into a database and analyzed statistically using Statistical Package for Social Sciences (SPSS) software (version 16.0). Percentages and frequencies were used to interpret the sample characteristics of the respondents. Chi-square and Fisher exact tests were used to estimate differences between independent variables and NHIS enrollment. Simple and multiple regression models were used to estimate the association between independent variables and enrollment in NHIS. Based on Gujarati and Porter [19], the multiple regression model was developed as follows.

1 $${\text{P}} = \upalpha_{0} + \upalpha_{ 1} {\text{TOR}} + \upalpha_{ 2} {\text{R}} + \upalpha_{ 3} {\text{MS }} + \upalpha {}_{ 4}{\text{WE }} + \upalpha_{ 5} {\text{MOA }} + \upalpha_{ 6} {\text{EEH}} + \upalpha_{ 7} {\text{OD }} + \upepsilon$$

P = probability of enrolling in NHIS. TOR, R, MS, WE, MOA, EEH, and OD refer to the type of respondents, religion, marital status, work experience, membership of workers association, exposure to electrical hazards and occupational diseases, respectively. Also, α = parameters to be estimated; ε = error term. Due to the use of multiple regression, we were compelled to choose different independent variables. The test results were considered significant at a probability value of 0.05 or less.

### Results

#### Sample characteristics of the respondents

About 63% of the respondents were welders. Approximately 41% were master welders. It was revealed that 62.9% were males. Also, 34.6% were aged 49 years or below, 48.3% had a secondary level of education and 55.1% were married. Approximately 32% had worked for more than 13 years and 92% received a monthly income of less than GH¢1000.00 ($1 was equivalent to GH¢4.4 as of the time of the survey). Approximately 17% were members of workers association, 99% were exposed to biological hazards, 64% were exposed to welding light, 62% were exposed to electrical hazards and 99% were exposed to fire hazards. About 39% were exposed to occupational injuries in 2016. Further, 61% had suffered from occupational diseases in 2016. The study revealed a statistically significant difference between type of respondent (p = 0.027), marital status (p = 0.037), religion (p = 0.010), work experience (p = 0.003), membership of workers association (p = 0.003), exposure to electrical hazards (p = 0.049) and suffering from occupational diseases in 2016 (p = 0.001) in relation to NHIS enrollment (see Table 1).

**Table 1:** Socio-demographic and medical characteristics of the respondents by NHIS enrollment

Variable	Category	Enrollment in NHIS (n = 350)						p-value
		Yes		No		Total		
		n = 61	%(100)	n = 289	%	N = 350	%	
Type of enterprise	Welding	34	55.7	186	64.4	220	62.9	0.205
	Fish processing	27	44.3	103	35.6	130	37.1	
Type of respondent	Master	33	54.1	111	38.4	144	41.1	0.027*
	Apprentice	11	18.0	99	34.3	110	31.4	
	Employee	17	27.9	79	27.3	96	27.4	
Gender	Male	34	55.7	186	64.4	220	62.9	0.205
	Female	27	44.3	103	35.6	130	37.1	
Age (years)	49 or below	14	23.0	107	37.0	121	34.6	0.065
	50 or above	47	77.0	182	63.0	229	65.4	
Educational attainment	No formal education	15	24.6	50	17.3	65	18.6	
	Basic education	8	13.1	71	24.6	79	22.6	0.096
	Secondary education	34	55.7	135	46.7	169	48.3	
	Tertiary	4	6.6	33	11.4	37	10.6	
Marital status	Married	41	67.2	152	52.6	193	55.1	0.037*
	Single	20	32.8	137	47.4	157	44.9	
Religion	Christian	53	86.9	271	93.8	324	92.6	0.010*
	Non-Christian (Islam/traditionalist/no religion)	8	13.1	18	6.2	26	7.4	
Work experience (years)	Below 2	6	9.8	14	4.8	20	5.7	
	2–4	6	9.8	55	19	61	17.4	
	5–7	5	8.2	63	21.8	68	19.4	
	8–10	9	14.8	58	20.1	67	19.1	0.003*
	11–13	4	6.6	17	5.9	21	6.0	
	Above 13	31	50.8	82	28.4	113	32.3	
Total income/allowance per month (GH¢)	< 1000	54	88.5	268	92.7	322	92.0	
	1000 or above	7	11.5	21	7.3	28	8.0	0.198
Membership of workers association	Yes	21	34.4	50	17.3	71	20.3	0.003*
	No	40	65.6	239	82.7	279	79.7	
Exposure to biological hazards at the work place	Yes	61	100	287	99.3	348	99.4	0.515
	No	0	0.0	2	0.7	2	0.6	
Exposure to welding light	Yes	32	52.5	184	63.7	216	61.7	0.102
	No	29	47.5	105	36.3	134	38.3	
Exposure to electrical hazards?	Yes	27	79.4	115	61.8	142	64.5	0.049*
	No	7	20.6	71	38.2	78	35.5	
Exposure to fire hazards	Yes	61	100	286	99	347	99.1	0.424
	No	0	0.0	3	1.0	3	0.9	
Did you experience an occupational accident which led to an injury in the year 2016?	Yes	24	39.3	112	38.8	136	38.9	0.932
	No	37	60.7	177	61.2	214	61.1	
Did you suffer from occupational diseases in the year 2016?	Yes	50	82.0	162	56.1	212	60.6	0.001*
	No	11	18.0	127	43.9	138	39.4	

* p-value is significant at 0.05 or less

#### Enrollment rate of NHIS

**Fig. 1:**
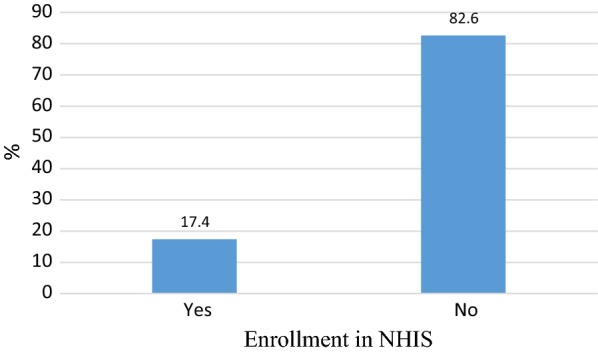
Enrollment rate of NHIS among informal sector workers

The result indicated that 17.4% of the respondents had enrolled in NHIS. This suggests a low enrollment in NHIS among the participants (see Fig. 1).

#### Factors associated with enrollment in NHIS

The variables that showed a statistically significant difference with NHIS enrollment in the Chi-square and Fisher exact test analyses were further subjected to simple and multiple regression models with the rationale of estimating their likelihood of influencing NHIS enrollment. In a simple regression model, the study found that apprentices were 0.676 times significantly less likely to enroll in NHIS compared with their masters (OR = 0.676, CI 0.284–0.576, p = 0.009). Non-Christian participants were 0.922 times significantly less likely to enroll in NHIS (OR = 0.922, CI 0.451–2.513, p = 0.003). Participants who had worked between 5 and 7 years were 5.4 times significantly more likely to enroll in NHIS (OR = 5.400, CI 1.442–20.226, p = 0.012). Respondents who were members of workers association were 2.509 times significantly more likely to enroll in NHIS (AOR = 2.509, CI 1.364–4.618, p = 0.03). Those who were exposed to electrical hazards were 1.8 times significantly more likely to enroll in NHIS (OR = 1.8, CI 1.102–3.05, p = 0.045). Participants who had suffered from occupational diseases were 2.2 times significantly more likely to enroll in NHIS (OR = 2.2, CI 1.25–4.256, p = 0.001).

In a multiple regression analysis, we found that apprentices were 0.72 times significantly less likely to enroll in NHIS than their masters (AOR = 0.72, CI 0.353–1.056, p = 0.005). Non-Christians were 0.726 times significantly less likely to enroll in NHIS (AOR = 0.726, CI 0.067–2.503, p = 0.011) Respondents who had worked between 5 and 7 years were 13.159 times significantly more likely to enroll in NHIS than their respective counterparts (OR = 13.159, CI 1.135–152.596, p = 0.039). Participants who were exposed to electrical hazards were 2.93 times significantly more likely to enroll in NHIS (OR = 2.93, CI 1.56–5.10, p = 0.013). Respondents who had suffered from occupational diseases were 2.75 times significantly more likely to enroll in NHIS (OR = 2.75, CI 1.743–5.17, p = 0.001) (see Table 2).

**Table 2:** Factors associated with enrollment in NHIS among informal sector workers

Variables	Simple regression model OR (95% CI)	p-value	Multiple regression model AOR (95% CI)	p-value
Type of respondent				
Master (ref)	1.00		1.00	
Apprentice	*0.676 (0.284*–0.576)*	0.009*	*0.72 (0.353*–1.056)*	0.005*
Employee	1.382 (0.720–2.653)	0.332	1.42 (1.13–3.201)	0.12
Marital status				
Married (ref)	1.00		1.00	
Single	*1.848 (1.032*–*3.308)*	0.039*	1.411 (0.593–4.125)	0.219
Religion				
Christian (ref)	1.00		1.00	
Non- Christian	*0.922 (0.451*–*2.513)*	0.003*	*0.726 (0.067*–*2.503)*	0.011*
Work experience				
Below 2 years (ref.)	1.00		1.00	
2 to 4 years	*3.929 (1.098*–*14.054)*	0.035*	*11.727 (0.998*–*137.824)*	0.05*
5 to 7 years	*5.400 (1.442*–*20.226)*	0.012*	*13.159 (1.135*–*152.596)*	0.039*
8 to 10 years	2.762 (0.843–9.047)	0.093	8.767 (0.843–91.170)	0.069
11 to 13 years	1.821 (0.427–7.761)	0.417	4.827 (0.366–63.721)	0.232
Above 13 years	1.134 (0.400–3.213)	0.813	2.571 (0.274–24.117)	0.408
Membership of workers association				
Yes	*2.509 (1.364*–*4.618)*	0.03*	4.20 (1.56–7.75)	
No (ref)	1.00		1.00	0.121
Exposure to electrical hazards				
Yes	*1.8 (1.102*–*3.05)*	0.045*	*2.93 (1.56*–*5.10)*	0.013*
No (ref)	1.00		1.00	
Suffering from occupational diseases				
Yes	*2.2 (1.25*–*4.256)*	0.001*	*2.75 (1.743*–*5.17)*	0.001*
No (ref.)	1.00		1.00	

Italic values indicate significance of *p* value

*CI* confidence interval, *OR* odd ratio, *AOR* adjusted odd ratio

* p-value is significant at 0.05 or less

### Discussion

Being one of the first studies in Ghana, this study examined factors influencing NHIS enrollment among informal sector workers in Metropolitan Kumasi. We found that 17.4% of the participants had enrolled in NHIS which is similar to a Namibian study that reported a 17.5% coverage rate of NHIS [15]. It is also similar to a 17.4% coverage rate among Ghanaian female migrants [13] but lower than 35% national enrollment rate in 2017 [6]. The low enrollment rate could be attributed to poor quality of services offered to the insured and failure of the National Health Insurance Authority (NHIA) to intensify education on NHIS. The NHIA should, therefore, engage health insurance stakeholders to develop and implement activities to enforce compulsory enrollment to move the scheme towards universal coverage [20].

In this study, apprentices were significantly less likely to enroll in NHIS. The reason is that master welders are more likely to be wealthier than their apprentices and thus are more likely to enroll in the scheme [21]. Also, apprentices may either find it difficult getting money to pay for their enrollment fee or are unable to make time out of their busy schedules to enroll in NHIS. Thus, masters should encourage their apprentices to enroll in NHIS. The study found that non-Christian participants were less likely to enroll in NHIS compared with their Christian counterparts. This result could be due to limited funds, religious-cultural beliefs, and differences in health-seeking behavior [22]. The study found that respondents who had worked for 5–7 years were more likely to enroll in NHIS than those who had worked below 2 years. This difference in the likelihood of enrollment in NHIS could be due to the differences in financial status and the number of times being exposed to occupational hazards. We further observed that respondents who were exposed to electrical hazards and experienced occupational diseases were more likely to enroll in NHIS which may be subject to their perceived poor health status [20, 23].

### Conclusion

This study examined factors predicting NHIS enrollment among informal sector workers in Kumasi Metropolis of Ghana. The study found a low enrollment rate in NHIS among informal sector workers. Respondents who had worked between 5 and 7 years were significantly more likely to enroll in NHIS. Also, apprentices were less likely to enroll in NHIS. Participants who were exposed to electrical hazards and occupational diseases were more likely to enroll in NHIS. Non-Christian participants were less likely to enroll in NHIS. These findings are useful for increasing the NHIS enrollment among informal sector workers in Ghana.

## Limitation

As a result of the cross-sectional survey adopted, we were not able to establish causality between and among the study variables.

## Data Availability

The datasets used and/or analyzed during the current study are available from the corresponding author on reasonable request.

## Abbreviations

AIDS: Acquired Immunodeficiency Syndrome
HIV: Human Immunodeficiency Virus (HIV)
NHIA: National Health Insurance Authority
NHIS: National Health Insurance Scheme
SSNIT: Social Security and National Insurance Trust
SPSS: Statistical Package for Social Sciences
